# N-terminal nesprin-2 variants regulate β-catenin signalling

**DOI:** 10.1016/j.yexcr.2016.06.008

**Published:** 2016-07-15

**Authors:** Qiuping Zhang, Rose-Marie Minaisah, Elisa Ferraro, Chen Li, Lauren J. Porter, Can Zhou, Fang Gao, Junyi Zhang, Dipen Rajgor, Flavia Autore, Catherine M. Shanahan, Derek T. Warren

**Affiliations:** British Heart Foundation Centre of Research Excellence, Cardiovascular Division, King's College, SE5 9NU London, UK

**Keywords:** NE, nuclear envelope, ONM, outer nuclear membrane, INM, inner nuclear membrane, F-actin, filamentous actin, EDMD, Emery–Dreifuss muscular dystrophy, CHD, calponin homology domain, SR, spectrin repeat, LINC, Linker of nucleoskeleton and cytoskeleton, WB, Western blot, IF, immunofluorescence microscopy, IP, immunoprecipitation, ESC, embryonic stem cells, VSMC, human vascular smooth muscle cell, HDF, human dermal fibroblast cell, HUVEC, human umbilical vein endothelial cells, Nesprin-2, Β-catenin, Cell-cell junctions, Scaffold protein

## Abstract

The spatial compartmentalisation of biochemical signalling pathways is essential for cell function. Nesprins are a multi-isomeric family of proteins that have emerged as signalling scaffolds, herein, we investigate the localisation and function of novel nesprin-2 N-terminal variants. We show that these nesprin-2 variants display cell specific distribution and reside in both the cytoplasm and nucleus. Immunofluorescence microscopy revealed that nesprin-2 N-terminal variants colocalised with β-catenin at cell-cell junctions in U2OS cells. Calcium switch assays demonstrated that nesprin-2 and β-catenin are lost from cell-cell junctions in low calcium conditions whereas emerin localisation at the NE remained unaltered, furthermore, an N-terminal fragment of nesprin-2 was sufficient for cell-cell junction localisation and interacted with β-catenin. Disruption of these N-terminal nesprin-2 variants, using siRNA depletion resulted in loss of β-catenin from cell-cell junctions, nuclear accumulation of active β-catenin and augmented β-catenin transcriptional activity. Importantly, we show that U2OS cells lack nesprin-2 giant, suggesting that the N-terminal nesprin-2 variants regulate β-catenin signalling independently of the NE. Together, these data identify N-terminal nesprin-2 variants as novel regulators of β-catenin signalling that tether β-catenin to cell-cell contacts to inhibit β-catenin transcriptional activity.

## Highlights

• N-terminal nesprin-2 variants display cell specific expression patterns.
• N-terminal spectrin repeats of nesprin-2 interact with β-catenin.
• N-terminal nesprin-2 variants scaffold β-catenin at cell-cell junctions..
• Nesprin-2 variants play multiple roles in β-catenin signalling.

## 1 Introduction

Nesprins are a family of spectrin repeat containing proteins that are encoded by four genes (*SYNE1-4*) [1], [2], [3], [4]. Nesprins-1 and -2 are highly complex and multiple variants arise due to alternative initiation and termination of the genes [5]. The giant nesprin-1 and -2 variants consist of an N-terminal paired calponin homology domain (CHD) that has been shown to bind filamentous actin (F-actin), a central rod region composed of numerous spectrin repeats and a C-terminal Klarsicht, ANC-1, SYNE Homology (KASH) domain that is required for the nuclear envelope (NE) localisation of these proteins [4], [6], [7]. To date, the best studied function of these proteins is at the NE, where smaller variants function to organise the inner nuclear membrane (INM) via interactions with lamins A/C and emerin [6], [8], whereas the nesprin giant variants reside on the outer nuclear membrane (ONM) and are components of the LInker of Nucleoskeleton to Cytoskeleton (LINC) complex. The LINC complex physically couples the ONM to the INM via interactions between the KASH domain of nesprins and the SUN domain of SUN1/2 in the perinuclear space [9], [10]. SUN1/2 span the INM and interact with lamins A/C [11], [12], thus forming a continuous biophysical network between the cytoskeleton and nucleoskeleton [10], [11], [12]. In addition to the giant nesprin-1 and -2 isoforms, nesprin variants that lack the KASH domain have been shown to localise to the cytoplasm and nucleoplasm [5], [13], [14], [15]. Although the functions of these KASH-less variants remain to be fully defined, they show tissue and cell specific expression patterns, suggesting nesprins are tailored for specific cellular functions.

Nesprins are comprised of multiple spectrin repeats that are proposed to mediate protein-protein interactions, however, our knowledge of nesprin binding partners remains limited [16]. At the INM, nesprin variants interact with lamins A/C, SUN1/2 and emerin [6], [12]. Mutations in these nesprin variants result in emerin mislocalisation, nuclear morphology defects and are associated with Emery–Dreifuss muscular dystrophy (EDMD), suggesting that nesprins perform a scaffolding role at the NE [1]. KASH-less variants also perform a scaffolding role in the nuclear interior and we have previously identified nesprin-2 as a nuclear ERK scaffold that tethers ERK1/2 at promyelocytic leukaemia nuclear bodies to regulate proliferation [14]. Importantly, several cytoplasmic binding partners have also been identified for nesprin-1 and -2 including the RNA binding proteins Dcp1a, Rck and Ago2, and meckelin, respectively [13], [17]. Moreover, nesprin-1 and -2 KASH-less variants localise to focal adhesions and actin/microtubule filaments, suggesting that the cytoplasmic KASH-less variants may perform a similar scaffolding role [5], [13]. Nesprin-2 has also been implicated in the WNT pathway that transfers signals from the plasma membrane to the nucleus via nuclear translocation of the transcription factor β-catenin [18], [19], [20], [21]. Both α- and β-catenin interact with spectrin repeats (SRs) toward the C-terminus of nesprin-2 giant to attenuate β-catenin signalling [22]. In addition to this direct interaction, nesprin-2 may also indirectly associate with β-catenin at the INM, where the nesprin-2 binding protein emerin interacts with β-catenin to facilitate its nuclear export [23].

In this study we investigate the role of recently identified N-terminal nesprin-2 variants that retain the CHD but lack the KASH domain. We show that these variants are novel components of cell-cell junctions, where they colocalise and interact with β-catenin. Importantly, these nesprin-2 variants anchor β-catenin to cell-cell junctions to negatively regulate β-catenin mediated transcriptional activity.

## 2 Materials and methods

### 2.1 Cell culture

Human bone osteosarcoma epithelial (U2OS), human umbilical vein endothelial cells (HUVEC), mouse C2C12 myoblast, human dermal fibroblast and human vascular smooth muscle cells were cultured as described previously [24], [25]. The following nesprin-2 siRNA oligomers targeting the N-terminus of the giant variant were used in this study: siN2CH2 (5′ AGGAAGACACCCAGAAGUU 3′), siN2CH3 (5′ CUUCAGAAUUGCAGAACAAUU 3′), siN2CH5 (5′ GCCUUCACGUGCUGGAUAAUU 3′), p220CH^Nesp2^ 3′UTR1 (5′ GAGAAUAGUCUGUGGAGAAUU 3′), p220CH^Nesp2^ 3′UTR2 (5′ GGAACGUAGUGGAGGAUAUU 3′), p380CH^Nesp2^ 3′UTR1 (5′ AUCGAAAGCCAGAGAGUAAUU 3′) and p380CH^Nesp2^ 3′UTR2 (5′ AGUCAGAGGUCAACAACAAUU 3′) (Dharmacon). C-terminal nesprin-2 siRNA designed to a region close to the KASH domain (siN2KASH) have been described previously [14]. Emerin smart pool siRNA oligomers from Dharmacon were used in this study. Transfection of siRNA was performed using HiPerfect (Qiagen), as per manufacturer's instructions. DNA transfections of were performed with Fugene (Promega) as per manufacturer's instructions.

### 2.2 PCR and 3′UTR amplification

PCR for N-terminal nesprin-2 3′UTRs were performed using 3′UTR specific primer sets as described previously [5].

### 2.3 Nesprin constructs

The following N-terminal nesprin-2 fragments were cloned into pEGFP-C1 vector (Clontech): ABDN2 (amino acids 1-531). The CHDN2 (amino acids 1-278) fragment was cloned into the pCMV-Tag vector (Agilent Technologies). The SR 1-3 region (amino acids 279-531) was cloned into the pGEX4T-1 (Amersham) and pCMV-Tag (Agilent Technologies) vectors.

### 2.4 Calcium switch assay

Cells were grown to 80–100% confluency and serum starved overnight. Next day, cells were incubated with 4 mM EGTA in calcium free media for 1 h to promote cadherin mediated cell-cell junction disassembly. Junction re-assembly was promoted by incubating cells in media containing 1.8 mM calcium for 1 h. Cells were fixed and processed for immunofluorescence microscopy.

### 2.5 Western blot analysis, antibodies and immunofluorescence microscopy

Cell lysates were run on 5% or 8% polyacrylamide gels and subjected to Western blotting as described previously [6]. Antibodies used for Western blot, confocal immunofluorescence microscopy (IF) and immunoprecipitation were; GFP (ab290), GFP-Sepharose (ab69314) (Abcam), Vinculin (Sigma), Emerin (VP-E602) (Vector Labs), lamin A/C (sc-6215) (Santa Cruz), total β-catenin, active β-catenin clone 8E7 (05-665) (Millipore), nesprin-2 CH3 and nesprin-2 N3 (Immune Systems). N2CH3 peptide blocking experiments were performed as described previously using the peptide KRDLDELKDHLQL (Immune Systems) [6]. Filamentous actin was observed by IF using Rhodamine phalloidin (Invitrogen). Secondary antibodies for WB were horseradish peroxidase-conjugated anti mouse (NA931) or anti rabbit (NA94V) antibodies from GE Healthcare. ECL chemiluminescent kit (RPN2132, GE Healthcare) was used for detection according to manufacturer's instructions. Invitrogen anti-mouse Alexa fluor 568 (A11031) and anti-rabbit Alexa fluor 488 (A11034) were used as IF secondary antibodies. For IF cells were cultured on cover slips, fixed in 4% paraformaldehyde (Sigma), permeabilised in 0.5% NP-40 (Sigma) and processed as described previously [6]. All images were captured at 63 × magnifications using a Leica SP5 laser scanning confocal microscope.

### 2.6 Immunoprecipitation, GST pull-downs and subcellular fractionations

Subcellular fractionations were performed as described previously [14]. GST expression, purification and pull-down assays were performed as described previously [14]. For immunoprecipitation (IP), U2OS cells were transfected with either GFP or GFP-ABDN2 and incubated overnight. Cells were processed for IP as described previously [14]. GFP was immunoprecipitated by incubating with anti-GFP coated Sepharose beads for 2 h at 4 °C. Beads were washed three times in IP buffer before bound proteins were eluted in sample buffer, as described previously [14]. Coomassie staining was performed using the Bio-Safe™ Coomassie stain (BIORAD) as per manufacturer's instructions.

### 2.7 Luciferase assays

U2OS cells were seeded onto a 6 well plate at a density of 2.5×10^5^ cells per well. Next day cells were transfected with mixtures of 1 µg TOP-FLASH or FOP-FLASH, 0.1 µg TK Renilla and 1 µg of GFP, GFP-ABDN2, FLAG or FLAG-SR 1-3 using Fugene (Promega). Cells were incubated overnight. For analysis of siRNA on transcriptional activity the TOP-FLASH or FOP-FLASH and TK Renilla mix was added directly to siRNA transfection mixture containing HiPerfect (Qiagen). Cells were incubated for 48 h. Luciferase and Renilla activities were assayed using the Dual-Glo® Luciferase assay system (Promega) as per manufacturer’s instructions. Control Luciferase activities were assigned a value of 1.

### 2.8 Statistical analysis

Results are presented as mean +/− SEM. For comparison of siRNA knockdown groups paired Student's *t*-tests or one way ANOVA with Bonferroni's post-test were performed.

## 3 Results

### 3.1 Cell specific distribution of nesprin-2 variants

**Fig. 1:**
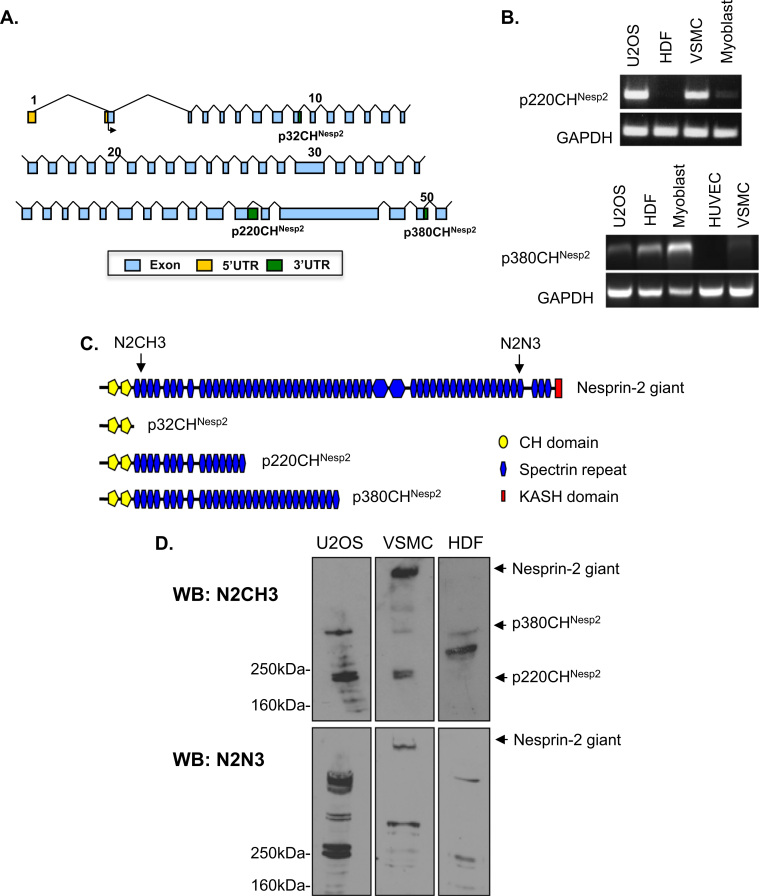
Cell type specific expression of N-terminal nesprin-2 variants. A) Schematic representation of the genomic organisation of 5′ and 3′ UTRs encoding the N-terminal variants of nesprin-2 N-terminus. B) PCR analysis of cDNA derived from U2OS, dermal fibroblast (HDF), vascular smooth muscle (VSMC), C2C12 myoblast and human umbilical vein endothelial (HUVEC) cells for p220CH^Nesp2^ and p380CH^Nesp2^ 3′UTRs. C) Schematic representation of nesprin-2 N-terminal variant structure and N-terminal (N2CH3) and C-terminal (N2N3) nesprin-2 antibody epitope regions. D) WB of U2OS, VSMC and HDF whole cell lysates separated on 5% polyacrylamide gels.

Recently, 3′UTRs encoding KASH-less N-terminal nesprin-2 variants (p220CH^Nesp2^ and p380CH^Nesp2^) were identified by EST data base searches (Fig. 1A). These 3′UTRs display tissue specific expression patterns [5]. To describe the cell specificity of these 3′UTRs, we performed PCR analysis and we show that p220CH^Nesp2^ is abundant in human bone osteosarcoma epithelial (U2OS) and vascular smooth muscle cells (VSMC) but absent in human dermal fibroblast (HDF) and mouse C2C12 myoblast cells. The p380CH^Nesp2^ variant was abundant in U2OS, HDF and myoblast cells, but lacking in human umbilical vein endothelial cells (HUVEC) and VSMCs (Fig. 1B). Western blot analysis (WB) was performed on whole cell lysates using an antibody raised to the N-terminus of the nesprin-2 giant (N2CH3) (Fig. 1C). To confirm the specificity of the N2CH3 antibody we performed peptide blocking experiments and show that the activity of the antibody is efficiently blocked by the target sequence on WB (Supplementary Fig. 1A). In agreement with the PCR data, we show that U2OS cells possess both the p220CH^Nesp2^ and p380CH^Nesp2^ variants whereas VSMCs and HDFs possess either the p220CH^Nesp2^ or p380CH^Nesp2^ variant, respectively (Fig. 1D). Importantly, using the N2CH3 antibody and a C-terminal nesprin-2 antibody (N2N3) we show that the nesprin-2 giant is highly abundant in VSMCs but was not detectable in U2OS and HDF cells tested (Fig. 1C and D). As previous studies have shown that the nesprin-2 giant is present in HDF cells at low levels, we performed subcellular fractionation experiments to concentrate the nuclear proteins [26]. WB revealed that nesprin-2 giant was weakly present in HDF nuclear fractions. Importantly, nesprin-2 giant was not detected in U2OS nuclear fractions, further confirming that U2OS cells lack nesprin-2 giant (Supplementary Fig. 1B).

**Fig. 2:**
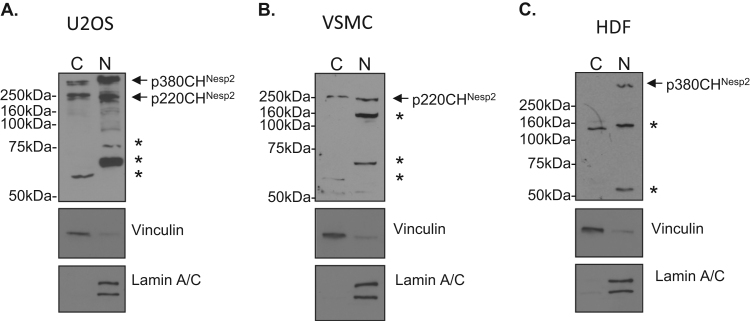
N-terminal nesprin-2 variants reside in the cytoplasm and nucleus. WB of U2OS, VSMC and HDF of cytoplasmic (C) and nuclear (N) fractions separated on 8% polyacrylamide gels. * mark unidentified nesprin variant bands.

Subcellular fractionation of U2OS cells demonstrated that p220CH^Nesp2^ and p380CH^Nesp2^ reside in both the cytoplasmic and nuclear fractions (Fig. 2A). In addition, smaller unknown nesprin-2 bands were observed in the cytoplasmic (55 kDa) and nuclear (60 and 70 kDa) fractions (Fig. 2A). The p220CH^Nesp2^ variant was also detected in both nuclear and cytoplasmic fractions in VSMCs (Fig. 2B), however, the p380CH^Nesp2^ variant was nuclear in HDF cells (Fig. 2C). In all cell types tested, unknown variants were detected, suggesting that our knowledge of nesprin-2 variants remains incomplete (Fig. 2A–C).

### 3.2 Nesprin-2 variants localise to cell-cell junctions and interact with β-catenin

**Fig. 3:**
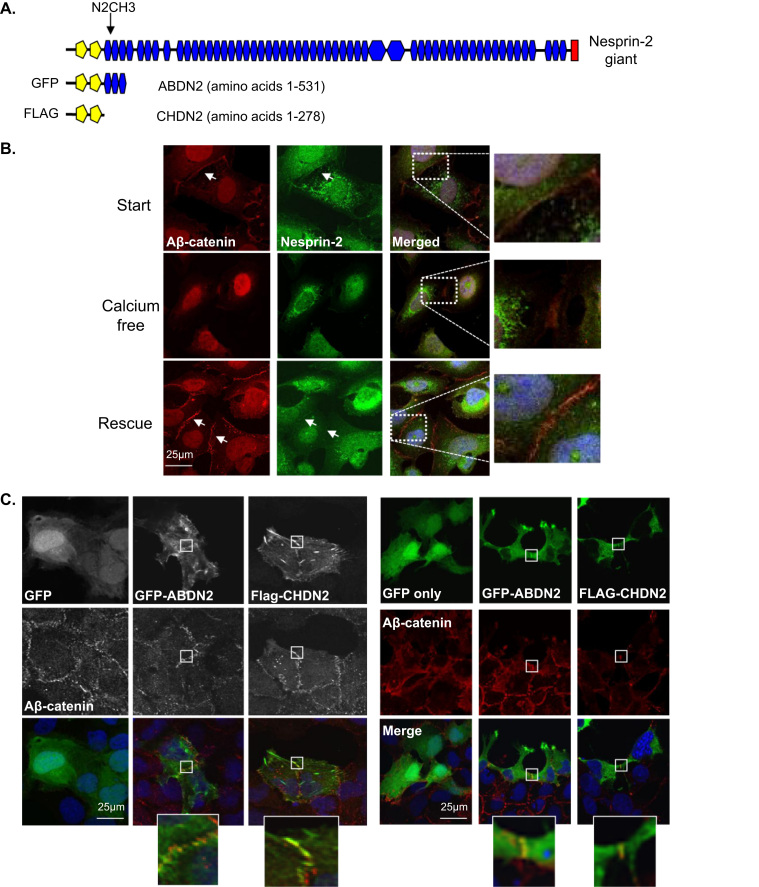
N-terminal nesprin-2 variants colocalise with β-catenin at cell-cell junctions. A) Schematic representation of nesprin-2 CH3 antibody epitope position and nesprin-2 constructs used. B) IF of nesprin-2 (CH3) (green), active β-catenin (Aβ-catenin) (red) and DAPI (blue) localisation in U2OS cells before, during and after calcium depletion. C) IF of GFP-ABDN2, Flag-CHDN2 (green) and active β-catenin (Aβ-catenin) (red) in U2OS (left panel) and fibroblast cells (right panel). Scale bar=25 µm.

Next, we employed confocal fluorescence microscopy (IF) to investigate the cellular localisations of these variants. IF demonstrated that the nesprin-2 antibody raised to the N-terminus of nesprin-2 giant (N2CH3) diffusely stained within the nucleus and at the sites of cell-cell contact at the cell periphery, where nesprin-2 colocalised with active β-catenin in U2OS cells (Fig. 3A and B). In contrast, no colocalisation with β-catenin was observed in HDF cells (Supplementary Fig. 2). To investigate the significance of nesprin-2 localisation at cell-cell contacts further, U2OS cells were grown in high or low calcium conditions to promote or inhibit cadherin mediated cell junction formation respectively. IF revealed that, U2OS cells in the presence of high calcium, displayed colocalisation of nesprin-2 and active β-catenin at cell-cell junctions, however, localisation of both nesprin-2 and β-catenin is rapidly lost from the plasma membrane when cells were switched to low calcium conditions to promote cadherin disassembly (Fig. 3B). Localisation of nesprin-2 and β-catenin at cell-cell contacts was rescued by replenishing calcium levels (Fig. 3B).

To further interrogate the localisation of nesprin-2 variants that retain the CHD but lack the KASH domain, we employed an overexpression strategy using an N-terminal nesprin-2 construct that possessed the CHD and the antibody binding region (amino acids 1-531) (Fig. 3A). IF demonstrated that the N-terminal fragment (GFP-ABDN2) colocalised efficiently with active β-catenin at cell-cell junctions in U2OS (Fig. 3C, left panel) and HDFs (Fig. 3C, right panel). Importantly a similar fragment of nesprin-1 failed to localise to cell-cell junctions and was predominantly nuclear, suggesting that cell-cell junction localisation is specific for nesprin-2 (Supplementary Fig. 3). To further define the requirements for cell-cell junction localisation, we next expressed the CHD region (amino acids 1-279) of nesprin-2. IF revealed that the CHD localised to cell-cell junctions, although some stress fibre staining was also observed (Fig. 3C).

**Fig. 4:**
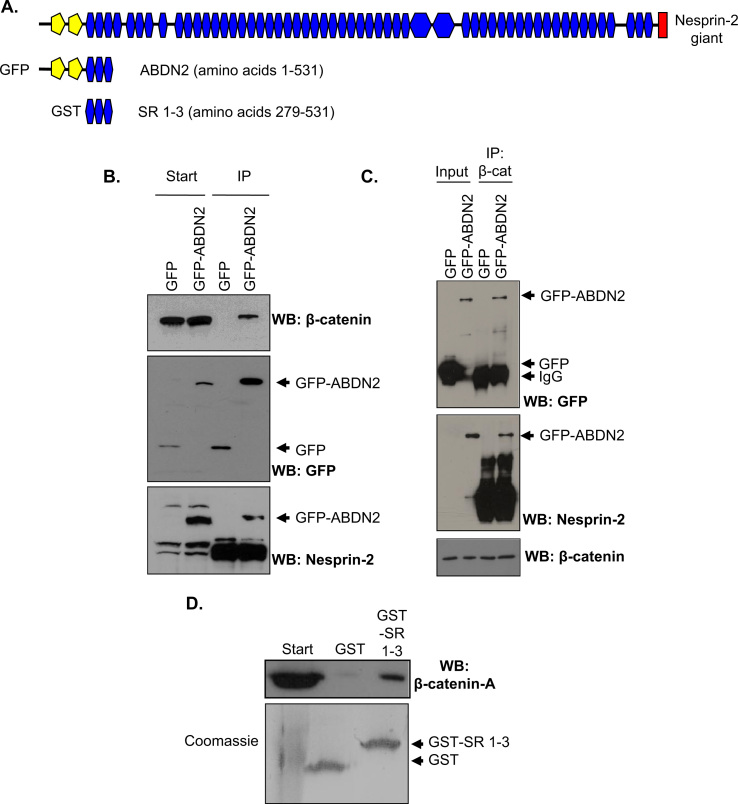
The N-terminus of nesprin-2 interacts with β-catenin. A) Schematic representation of the nesprin-2 constructs used. B) WB of GFP/GFP-ABDN2 immunoprecipitation. C) WB of β-catenin IP. D) WB of GST-alone and GST-SR 1-3 construct pull downs. GST-loading was shown by coomassie stain.

Next, we investigated whether nesprin-2 interacted with β-catenin by performing immunoprecipitation experiments. WB revealed that β-catenin was precipitated by the GFP-ABDN2 fragment but not GFP-alone (Fig. 4A and B). Conversely, the GFP-ABDN2 fragment was efficiently precipitated by β-catenin IP, confirming the nesprin-2 is a novel β-catenin interacting protein (Fig. 4C). Next, we mapped the β-catenin binding site by fusing the SR region of the ABDN2 construct (SR 1-3 containing amino acids 278-531) to GST (Fig. 4A). GST pull down assays confirmed that β-catenin was precipitated by GST-SR 1-3, but not GST alone (Fig. 4D), confirming that this spectrin repeat region interacts with β-catenin.

### 3.3 Nesprin-2 disruption induces cell-cell junction disassembly and augments β-catenin transcriptional activity

**Fig. 5:**
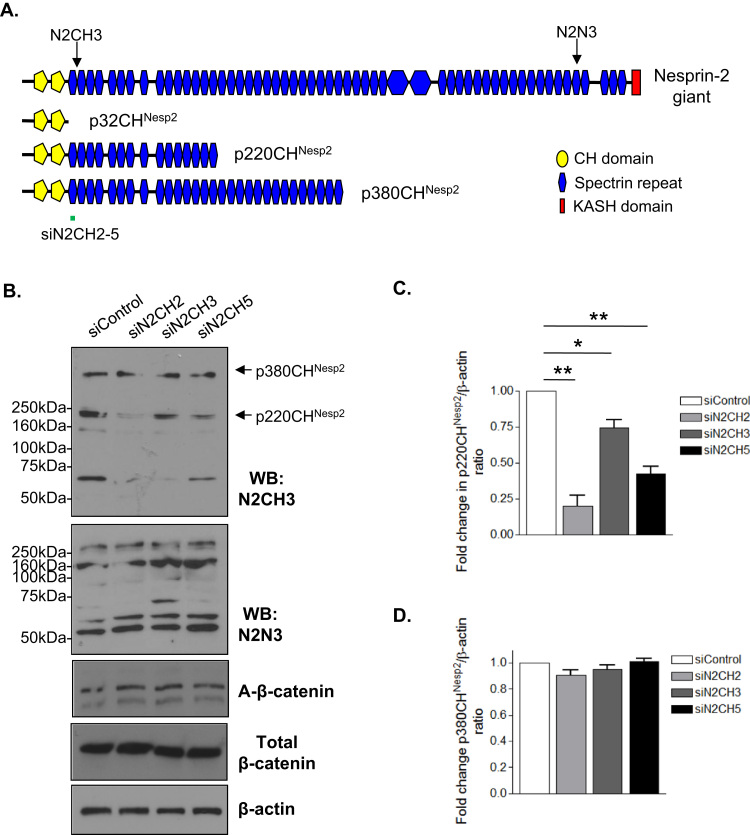
Validation of nesprin-2 depletion strategy. A) Schematic representation of nesprin-2 CH3 and N3 antibody epitopes and the region targeted by siRNA siN2CH2–5. B) WB of N-terminal (N2CH3) and C-terminal (N2N3) variants after control and nesprin-2 (siN2CH2/CH3/CH4) siRNA knockdown. Samples were separated on 8% polyacrylamide gels. Graphs show relative level of C) p220CH^Nesp2^ and D) p380CH^Nesp2^. Graphs represent combined data from 3 independent siRNA experiments for fold change in densitometry ratio (*p=<0.05, **p=<0.001).

We next investigated the impact of nesprin-2 disruption on β-catenin localisation by utilising a siRNA mediated knockdown strategy that targeted nesprin-2 variants containing the CHDs. U2OS cells were transfected with either control or nesprin-2 specific siRNA that targeted the N-terminus of p220CH^Neps2^ and p380CH^Nesp2^ (Fig. 5A). WB analysis confirmed knockdown of p220CH^Nesp2^ using 3 independent nesprin-2 specific siRNAs (Fig. 5B and C). Levels of the p380CH^Nesp2^ variant remained unaltered by our siRNA strategy (Fig. 5B and D), suggesting that p380CH^Nesp2^ is more stable than p220CH^Nesp2^. WB also revealed that protein levels of C-terminal variants that lack the siRNA target sequence remain unaltered (Fig. 5B). Importantly, levels of active β-catenin and total β-catenin remained unaltered by our nesprin-2 depletion strategy (Fig. 5B). To specifically target the p220CH^Nesp2^ and p380CH^Nesp2^ variants we designed siRNAs targeting the unique 3′UTRs, however, WB revealed that this strategy was unsuccessful and failed to deplete the p220CH^Nesp2^ and p380CH^Nesp2^ variants (Supplementary Fig. 4).

**Fig. 6:**
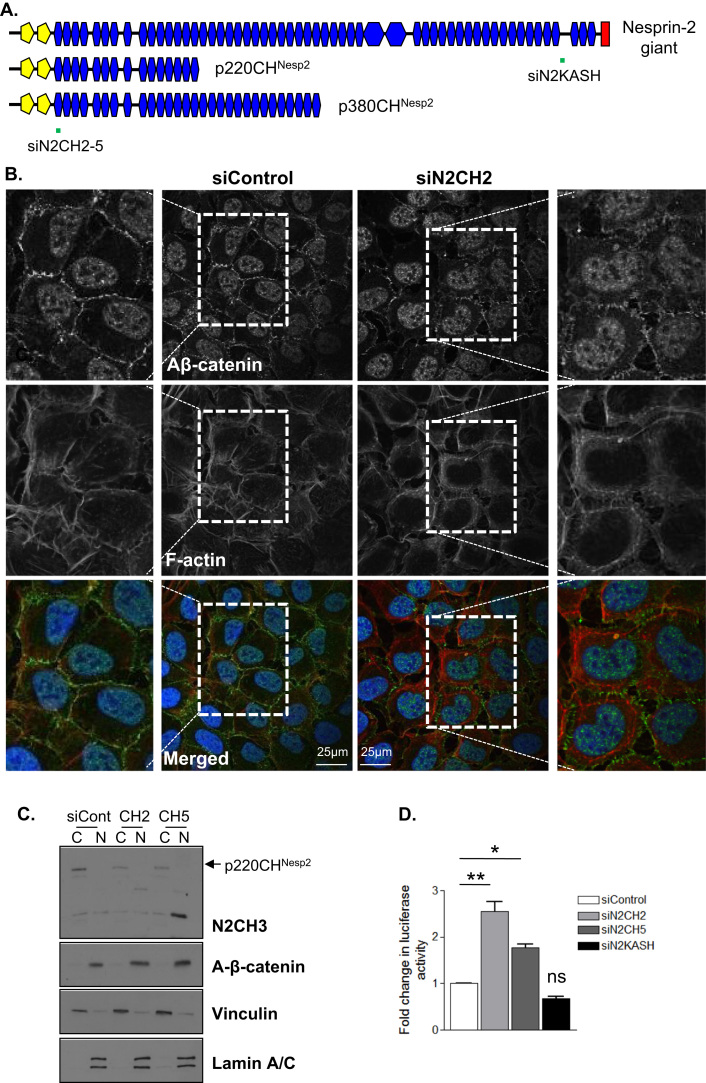
Nesprin-2 is required for β-catenin localisation at cell-cell junctions and negatively regulates β-catenin transcriptional activity. A) Schematic representation of siN2CH2/siN2CH5 and siN2KASH target regions. B) IF of active β-catenin (Aβ-catenin) (green), F-actin (red) and DAPI (blue) in control and nesprin-2 (siN2CH2) depleted U2OS cells. Scale bar=25 µm. C) WB of control, siN2CH2 and siN2CH5 cytoplasmic (C) and nuclear (N) fractions. D) TOP/FOP Luciferase assay of control, siN2CH2, siN2CH5 and siN2KASH depleted cells. Graph shows combined data from 3 independent experiments repeated in triplicate (*p=<0.05, **p<0.001).

IF was performed to observe whether nesprin-2 depletion altered β-catenin organisation in U2OS cells and revealed that nesprin-2 depleted cells displayed reduced staining of active β-catenin at cell-cell junctions compared to control cells (Fig. 6A and B and Supplementary Fig. 5A), suggesting that the p220CH^Nesp2^ variant tethers active β-catenin to the sites of cell-cell contact. Importantly, subcellular fractionation revealed that nesprin-2 depleted cells displayed increased levels of nuclear active β-catenin (Fig. 6C) and TOP-FLASH/FOP-FLASH luciferase assays confirmed that nesprin-2 depleted cells possessed augmented β-catenin transcriptional activity compared to control cells (Fig. 6D). In contrast, siRNAs targeting the C-terminus of nesprin-2 giant that the p380CH^Nesp2^ and p220CH^Nesp2^ variants lack, failed to alter luciferase activity (Fig. 6A and D), supporting the notion that N-terminal variants are responsible for localising β-catenin to cell-cell junctions. Next, we assessed the impact of overexpression of the β-catenin binding fragments of the N-terminal nesprin-2 variants on β-catenin signalling. However, TOP-FLASH/FOP-FLASH luciferase assays revealed that the β-catenin binding fragments had no impact on β-catenin transcriptional activity (Supplementary Fig. 5B).

### 3.4 β-catenin localisation at cell-cell junctions is independent of emerin

**Fig. 7:**
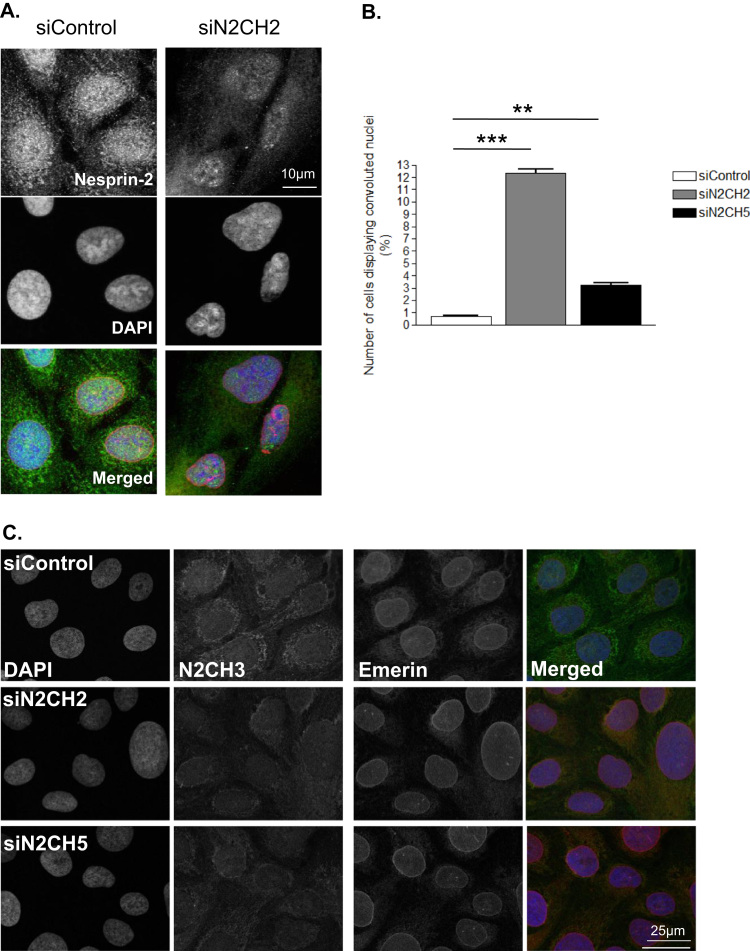
Nesprin-2 disruption alters nuclear morphology but not emerin localisation. A) IF of nesprin-2 (green), emerin (red), and DAPI (blue) staining of control and nesprin-2 depleted cells. Scale bar=10 µm. B) Quantification of number of control and nesprin-2 depleted (siN2CH2 and siN2CH5) cells displaying convoluted nuclei. Graph show combined data from 3 independent experiments counting 300 cells per group (**p=<0.001 and ***p=<0.0001). C) IF staining of DAPI (blue), N2CH3 (green) and emerin (red) in control and nesprin-2 depleted U2OS cells. Scale bar=25 µm.

**Fig. 8:**
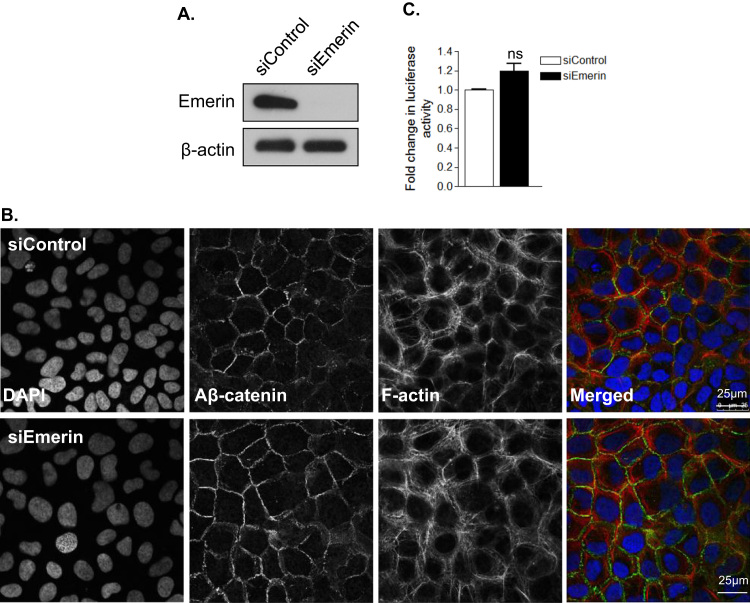
Emerin disruption does not impact on β-catenin localisation. A) WB confirming emerin knockdown. B) IF of active β-catenin (Aβ-catenin) (green), F-actin (red) and DAPI (blue) in control and emerin depleted U2OS cells. Scale bar=25 µm. C) TOP/FOP luciferase assay of control and emerin depleted cells. Graph shows combined data from 3 independent experiments repeated in triplicate.

Previous studies have shown that nesprin disruption triggers nuclear morphology defects, so we next performed IF to observe if our siRNA strategy altered nuclear morphology. Analysis revealed that control cells contained spherical nuclei, however, nesprin-2 depleted nuclei possessed a more convoluted morphology (Fig. 7A and B). Next, we performed IF to observe the localisation of the nesprin-2 interacting protein emerin and show that nesprin-2 depleted cells display normal NE emerin staining (Fig. 7C). As emerin has previously been implicated in β-catenin signalling, we further investigated whether changes in β-catenin signalling were due to impaired emerin function by performing emerin knockdown experiments. WB confirmed efficient emerin depletion in U2OS cells (Fig. 8A), however, β-catenin organisation and transcriptional activity remained unaltered in emerin depleted cells (Fig. 8B and C).

## 4 Discussion

Nesprins have emerged as signalling scaffold proteins that localise to multiple subcellular compartments, including the NE, cytoplasm and nucleoplasm [5], [6]. In this current study, we show that nesprin-2 N-terminal variants colocalise with β-catenin at cell-cell junctions. We show that a fragment containing the CHD and SR 1-3 region (ABDN2) was sufficient for both β-catenin binding and cell-cell junction localisation. Further mapping identified the N-terminal SRs 1-3 of nesprin-2 as a novel β-catenin binding region, although we did not rule out the possibility that the CHD and β-catenin also interact. We propose that the N-terminal nesprin-2 KASH-less variants tether β-catenin at cell-cell junctions and inhibit β-catenin transcriptional activity. In support of this notion, nesprin-2 depleted U2OS cells displayed loss of β-catenin from cell-cell contacts, accumulation of active β-catenin in the nucleus and augmented β-catenin transcriptional activity. Importantly, levels of active β-catenin remained unchanged in nesprin-2 depleted U2OS cells, suggesting that nesprin-2 depletion triggers redistribution of active β-catenin from cell-cell contacts to the nucleus. Our overexpression experiments show that N-terminal CHD containing nesprin-2 fragments localise to cell-cell contacts, colocalise with active β-catenin but did not alter β-catenin transcriptional activity. N-terminal nesprin-2 fragments that lack the CHD but retain the β-catenin binding site also failed to alter β-catenin transcriptional activity. This suggests that the association between active β-catenin and the N-terminal nesprin-2 variants at cell-cell contacts is stable, further experimentation is required to elucidate the functions/dynamics of these N-terminal nesprin-2 variants at cell-cell contacts.

We also demonstrate that the p220CH^Nesp2^ and p380CH^Nesp2^ variants, that contain the β-catenin binding domain, reside in both the nucleus and cytoplasm, raising the intriguing possibility that these KASH-less variants may shuttle between these compartments. However, further investigation is now required to clarify whether KASH-less nesprin-2 variants associate with and organise other components of the β-catenin pathway, as well as to identify the exact nesprin-2 variant. Our knockdown strategy efficiently depleted p220CH^Nesp2^ but not p380CH^Nesp2^ and presumably these two variants display differences in protein turnover as both contain the target sequence. This suggests that p220CH^Nesp2^ is potentially a good candidate for future investigation however, our siRNA strategy targeted multiple nesprin-2 variants so the possibility remains that an unidentified variant may localise to cell-cell contacts and regulate β-catenin signalling.

### 4.1 N-terminal nesprin-2 variants regulate β-catenin signalling independently of the NE

Nesprin-2 variants organise the NE and several recent studies have identified the NE as a novel regulator of β-catenin signalling [22], [23], [30]. Firstly, β-catenin interacts with the C-terminus of the nesprin-2 giant to positively regulate β-catenin signalling [22]. In addition, the nesprin-1/2 orthologue ANC1 regulates β-catenin signalling during neuronal development in *Caenorhabditis elegans* [30]. Due to the sequence identity between nesprin-2 variants, our knockdown strategy potentially targeted both KASH-less N-terminal and the KASH-containing nesprin-2 giant variants [5]. Importantly, we show that U2OS cells lack nesprin-2 giant and β-catenin transcriptional activity was enhanced by N-terminal nesprin-2 depletion in U2OS cells. These data suggest that the N-terminal nesprin-2 variants negatively regulate β-catenin transcriptional activity in U2OS cells and highlight the complexity of nesprin-2 function in regulating β-catenin signalling. Previous studies have also demonstrated that emerin interacts with both the C-terminal nesprin-2 variants and β-catenin at the INM to negatively regulate β-catenin mediated transcription [23]. KASH-containing nesprin-2 variants are essential for emerin organisation at the NE [6], however, emerin organisation was unaltered by depletion of N-terminal nesprin-2 variants in U2OS cells, suggesting that changes in β-catenin signalling induced by our siRNA strategy were NE independent. Furthermore, emerin depletion in U2OS cells failed to displace β-catenin from cell-cell junctions or alter β-catenin transcriptional activity, further suggesting that the N-terminal nesprin-2 variants regulate β-catenin signalling independently of the NE.

Despite our evidence showing that nuclear envelope function is not disrupted by our nesprin-2 siRNA strategy, nuclear morphology was altered by our approach. Previous studies have demonstrated that actomyosin, cell morphology and adhesion all contribute to defining nuclear morphology and potentially, in addition to disrupting cell-cell contacts, our nesprin-2 depletion strategy induced cytoskeletal reorganisation that altered nuclear morphology [27], [28]. However, the potential role of the nesprin-2 N-terminal variants in cytoskeletal organisation remains untested.

### 4.2 Nesprin-2 variants fine tune β-catenin signalling for cell specific functions?

Nesprin variants demonstrate complex tissue and cell-specific distributions [5], [29]. Nesprin giant variant expression is abundant in the majority of human tissues, except cardiac and skeletal muscle, which are enriched in shorter isoforms [29]. In addition, the nesprin-2 epsilon-1 and epsilon-2 variants are highly expressed in embryonic stem cells (ESC) and heart respectively [29]. Here, we confirm that the p220CH^Nesp2^ and p380CH^Nesp2^ variants display cell-specific expression, suggesting that nesprin function is tailored to specific cellular functions. In support of this, up regulation of nesprin variants and nesprin variant switching is observed in ESC, mesenchymal stem cell and skeletal muscle differentiation [26], [30], [31]. Nesprins have emerged as signalling scaffolds for the ERK and β-catenin pathways and these pathways exist in multiple cell types. Furthermore, recent evidence demonstrates the importance of the signalling scaffolding functions of the nesprin family during development, where the nesprin-1/2 orthologue ANC1 regulates β-catenin signalling during neuronal development in *C. elegans* [32]. However, we show that the nesprin-2 giant is not detectable in U2OS cells and potentially adaptation of nesprin variant expression may fine tune these pathways and facilitate cell-specific signalling. In support of this, we show that U2OS cells display high levels of the p220CH^Nesp2^, whereas fibroblasts lack the p220CH^Nesp2^ variant. In addition to changes in nesprin-2 variant expression, the p380CH^Nesp2^ variant displayed differential compartmentalisation between U2OS and HDF cells, therefore, differential nesprin variant expression/compartmentalisation may contribute to cell specific functions for nesprin-2 in β-catenin signalling. Further investigation is now required to clarify the cell-specific functions of nesprin variants in regulating β-catenin signalling.

## Conflicts of interest

The authors declare that no conflicts of interest exist.

## Sources of funding

This work was funded by a British Heart Foundation, United Kingdom (BHF) program grant to CMS (program Grant number RG/11/14/29056), a BHF IBSRF awarded to DTW (FS/11/53/29020) and a BHF project grant to QPZ (PG/11/58/29004).
