# Supplementary material for: N-terminal nesprin-2 variants regulate β-catenin signalling
Source: Exp Cell Res. 2016 Jul 15;345(2):168–79. doi: 10.1016/j.yexcr.2016.06.008 (PMC4948682; doi:10.1016/j.yexcr.2016.06.008)
Supplement: Supplementary file 1 — Supplementary material [file mmc1.pptx]

## Slide 1
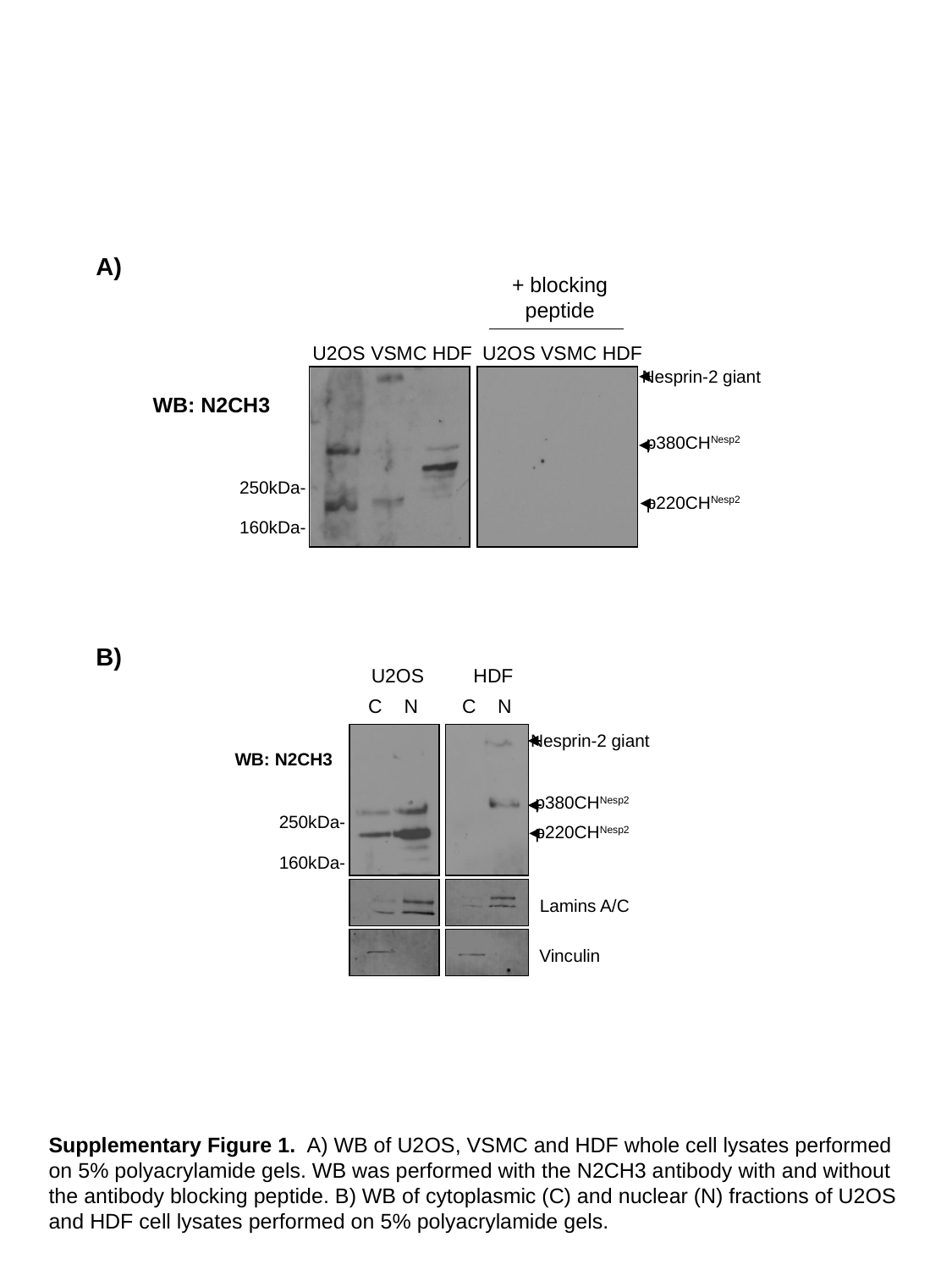

A)
+ blocking
peptide
U2OS VSMC HDF
U2OS VSMC HDF
Nesprin-2 giant
WB: N2CH3
p380CHNesp2
250kDa-
p220CHNesp2
160kDa-
B)
U2OS HDF
C N C N
Nesprin-2 giant
p380CHNesp2
p220CHNesp2
250kDa-
160kDa-
Lamins A/C
Vinculin
WB: N2CH3
Supplementary Figure 1. A) WB of U2OS, VSMC and HDF whole cell lysates performed on 5% polyacrylamide gels. WB was performed with the N2CH3 antibody with and without the antibody blocking peptide. B) WB of cytoplasmic (C) and nuclear (N) fractions of U2OS and HDF cell lysates performed on 5% polyacrylamide gels.

## Slide 2
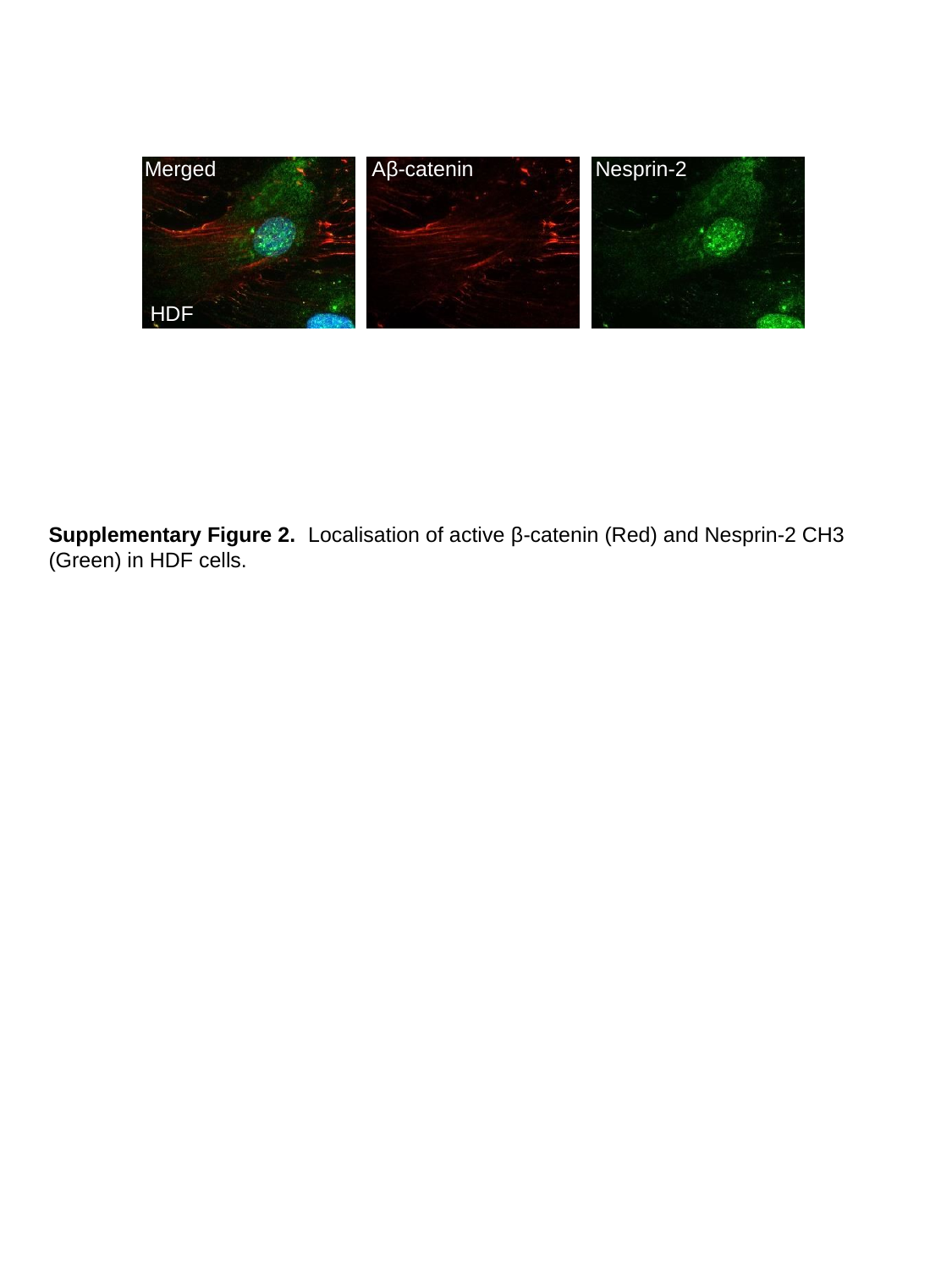

Merged
Aβ-catenin
Nesprin-2
HDF
Supplementary Figure 2. Localisation of active β-catenin (Red) and Nesprin-2 CH3 (Green) in HDF cells.

## Slide 3
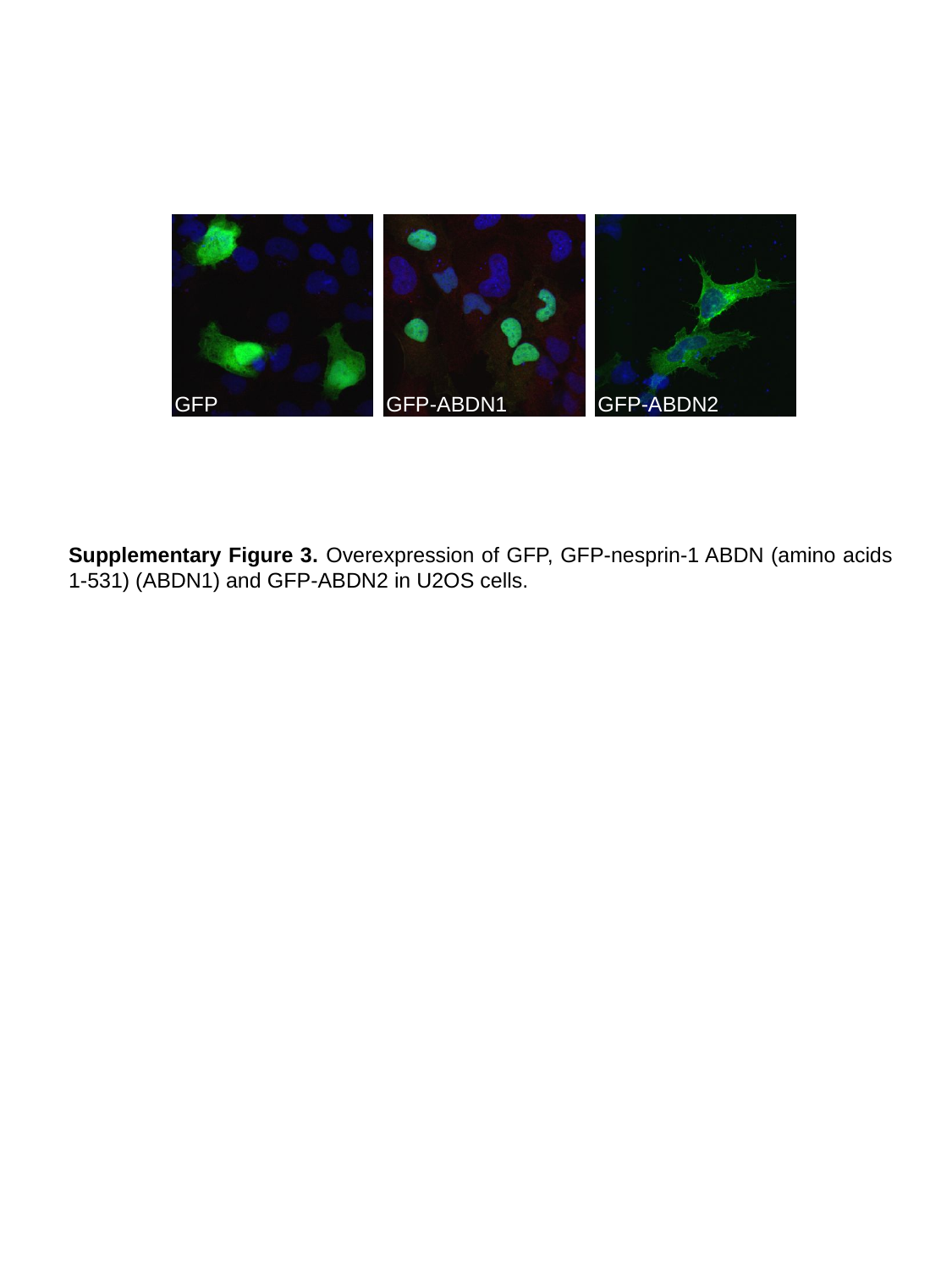

GFP
GFP-ABDN1
GFP-ABDN2
Supplementary Figure 3. Overexpression of GFP, GFP-nesprin-1 ABDN (amino acids 1-531) (ABDN1) and GFP-ABDN2 in U2OS cells.

## Slide 4
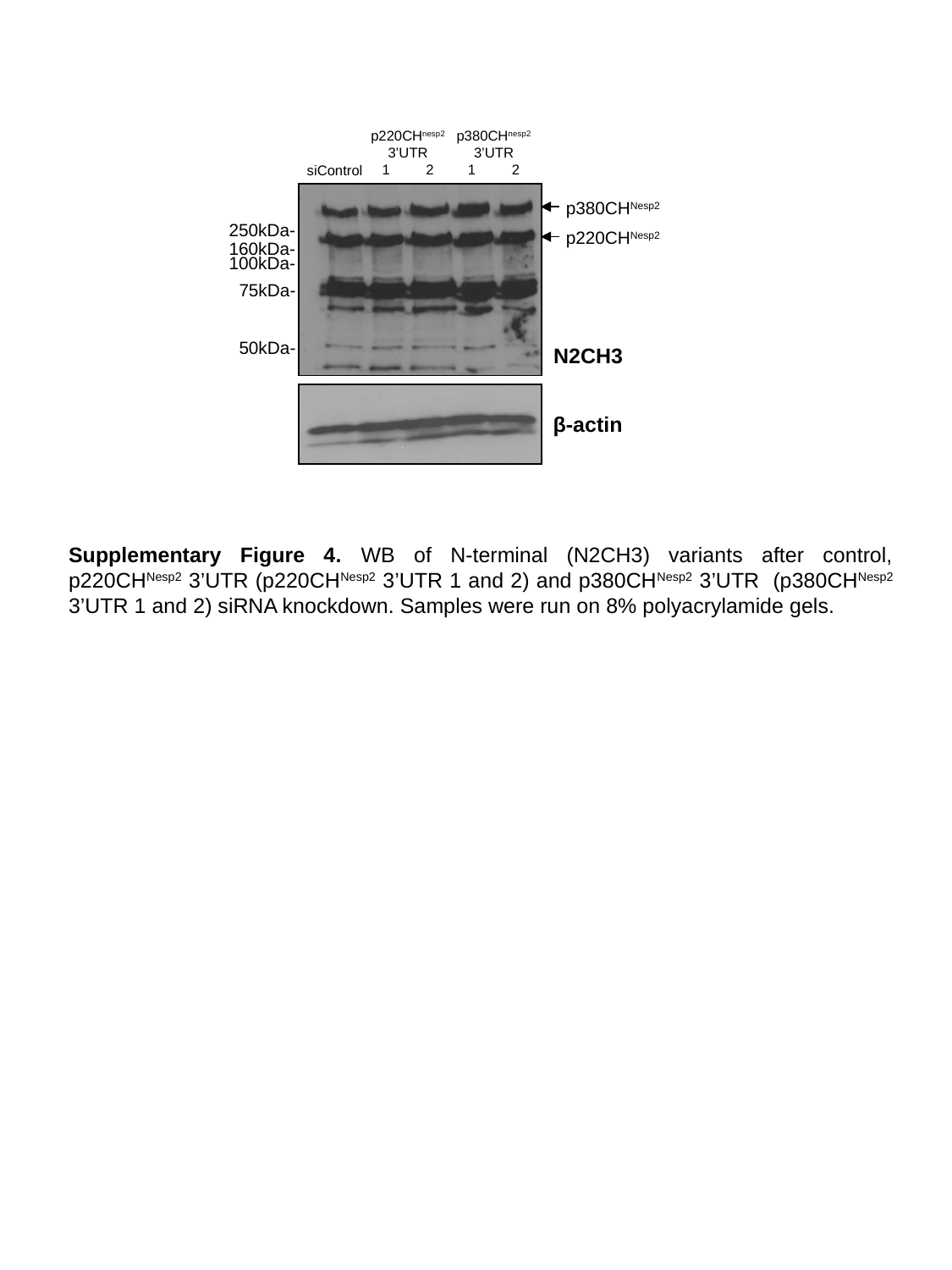

p220CHnesp2
3’UTR
1 2
p380CHnesp2
3’UTR
1 2
siControl
p380CHNesp2
250kDa-
160kDa-
100kDa-
75kDa-
50kDa-
p220CHNesp2
N2CH3
β-actin
Supplementary Figure 4. WB of N-terminal (N2CH3) variants after control, p220CHNesp2 3’UTR (p220CHNesp2 3’UTR 1 and 2) and p380CHNesp2 3’UTR (p380CHNesp2 3’UTR 1 and 2) siRNA knockdown. Samples were run on 8% polyacrylamide gels.

## Slide 5
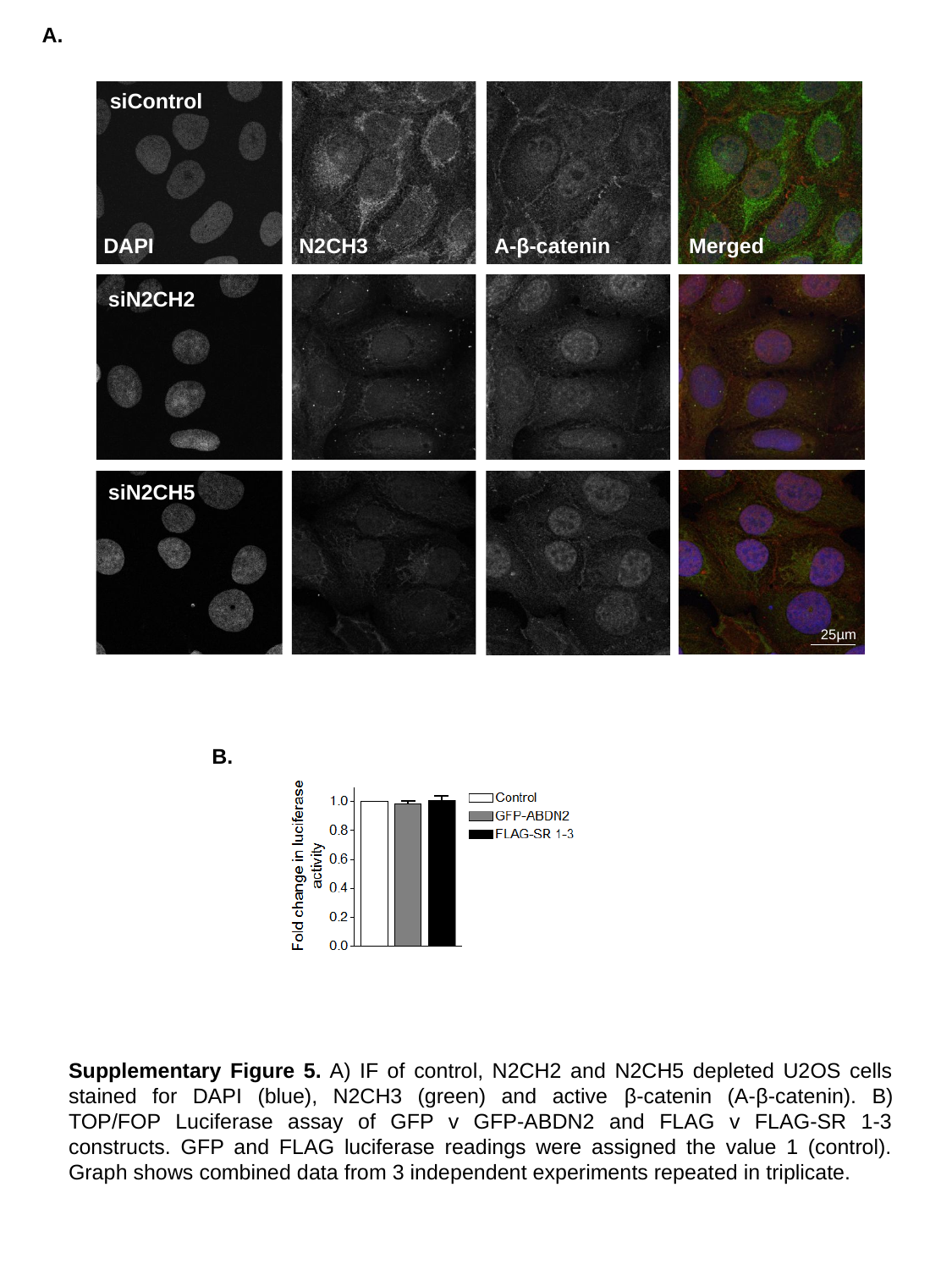

A.
siControl
DAPI
N2CH3
A-β-catenin
Merged
siN2CH2
siN2CH5
25µm
B.
Supplementary Figure 5. A) IF of control, N2CH2 and N2CH5 depleted U2OS cells stained for DAPI (blue), N2CH3 (green) and active β-catenin (A-β-catenin). B) TOP/FOP Luciferase assay of GFP v GFP-ABDN2 and FLAG v FLAG-SR 1-3 constructs. GFP and FLAG luciferase readings were assigned the value 1 (control). Graph shows combined data from 3 independent experiments repeated in triplicate.
